# Association of lymphocyte subsets with efficacy and prognosis of immune checkpoint inhibitor therapy in advanced non-small cell lung carcinoma: a retrospective study

**DOI:** 10.1186/s12890-022-01951-x

**Published:** 2022-04-28

**Authors:** Yi Yan, Xinyan Wang, Chenan Liu, Junmei Jia

**Affiliations:** Department of Oncology, The First Hospital of Shanxi Medical University, Taiyuan, Shanxi China

**Keywords:** Immune checkpoint inhibitors (ICIs), Biomarkers, Non-small cell lung cancer (NSCLC), Peripheral blood lymphocyte subsets

## Abstract

**Background:**

Immune checkpoint inhibitors (ICIs) have achieved promising effects in patients with non-small cell lung cancer (NSCLC). However, not all patients with NSCLC benefit from immunotherapy. There is an urgent need to explore biomarkers that could predict the survival outcomes and therapeutic efficacy in advanced NSCLC patients treated with immunotherapy. In this study, we aimed to assess the changes in peripheral blood lymphocyte subsets and their association with the therapeutic efficacy and clinical prognosis of advanced NSCLC patients treated with immunotherapy.

**Methods:**

A total of 276 patients with advanced NSCLC were enrolled. Peripheral blood lymphocyte subsets including CD4^+^ T cells, CD8^+^ T cells, CD4^+^/CD8^+^ ratio, NK cells, Tregs and B cells were collected before any treatment, before immunotherapy or chemotherapy, and after 4 cycles of immunotherapy or chemotherapy. T-test was used to analyze the factors influencing lymphocyte subsets and their changes before and after therapy. Logistic regression was used to plot ROC curves and analyze the relationship between lymphocyte subsets and therapeutic efficacy. Log-rank test and Cox regression model were used to evaluate the relationship between lymphocyte subsets and progression-free survival (PFS).

**Results:**

Gender, distant metastasis, and EGFR mutation status are known to affect the proportion of peripheral blood lymphocyte subsets in patients with advanced NSCLC. The proportions of CD4^+^ T cells, CD8^+^ T cells, Tregs and B cells were found to decrease after chemotherapy as compared to the baseline. The proportion of CD4^+^ T cells, CD8^+^ T cells, CD4^+^/CD8^+^ ratio, NK cells and Tregs were higher after immunotherapy than after chemotherapy. Compared to the baseline, the effective group showed significant increase in the proportions of CD4^+^ T cells, CD4^+^/CD8^+^ ratio, NK cells and Tregs, and the number of CD8^+^ T cells was significantly lower in the peripheral blood after 4 cycles of immunotherapy. On the contrary, the ineffective group did not show any significant differences in the above parameters. Baseline CD4^+^ T cells and NK cells were independent predictors of immunotherapy efficacy and PFS. Baseline Tregs were independent predictor of immunotherapy efficacy.

**Conclusion:**

Immune checkpoint inhibitors induced changes in the proportion of peripheral blood lymphocyte subsets in patients that responded well to immunotherapy. The levels of the different lymphocyte subsets could serve as valuable predictive biomarkers of efficacy and clinical prognosis for NSCLC patients treated with immunotherapy.

**Supplementary Information:**

The online version contains supplementary material available at 10.1186/s12890-022-01951-x.

## Introduction

Lung cancer is one of the most common malignancy and the leading cause of cancer death worldwide [1]. Non-small cell lung cancer (NSCLC) accounts for about 80% of primary lung malignancies. Due to the availability of improved therapeutics, the two-year relative survival rate of NSCLC patients has increased from 30 to 36% between 2010 and 2016 [2]. The use of immune checkpoint inhibitors (ICIs) has achieved promising efficacy and has significantly improved the clinical outcomes for NSCLC patients [3]. However, not all NSCLC patients benefit from immunotherapy. Moreover, around 4–29% of advanced NSCLC patients treated with mono-immunotherapy, have been reported to experience hyper-progression [4].

There is an urgent need to explore predictive biomarkers of efficacy and survival outcomes after immunotherapy treatment in advanced NSCLC patients, which could enable early intervention of the ongoing treatment strategies. To date, the expression of programmed cell death ligand 1 (PD-L1) has demonstrated a significant predictive role [5]. However, other biomarkers such as tumor mutation burden (TMB) and lung immune prognostic index (LIPI) have yielded conflicting results [6]. Therefore, the development of a dynamic, non-invasive and convenient approach to monitor the efficacy of ICIs is the main focus of the current research.

The theory of cancer immunoediting, including immune elimination, homeostasis and escape, states that immunity closely relates to the occurrence and progression of cancer, and is the basis of the anti-cancer effects of ICIs [7]. Lymphocytes are essential components of the human immune system. Many of the current studies focus on the tumor-infiltrating lymphocytes (TILs) in the tumor microenvironment, a heterogeneous population including two distinct pools of effector (CD4^+^ T cells, CD8^+^ T cells, NK cells) and suppressor (Tregs) phenotypes. These lymphocytes have been shown to be correlated with the efficacy of immunotherapy [8]. However, immunoediting occurs not only locally but also systemically to a large extent. Intense lymphocyte trafficking is observed in NSCLC patients and the phenotype of immune response at the organismal level is also observed in the blood [9]. Thus, we chose to study the peripheral blood lymphocyte subsets rather than the local immune status. Though numerous studies have shown that lung cancer patients have lower levels of CD4^+^ T cells, CD4^+^/CD8^+^ ratio, NK cell levels, and a higher regulatory T cells (Tregs) levels as compared to the healthy population [10, 11], the prognostic value of peripheral blood lymphocyte subsets in predicting immunotherapy efficacy in advanced NSCLC patients has not been demonstrated.

Our current work aims to analyze the impact of the clinicopathological characteristics on the peripheral blood lymphocyte subsets in advanced NSCLC patients in the real world. We sought to assess the dynamic changes in the levels of peripheral blood lymphocyte subsets before and after immunotherapy, and ultimately determine their correlation with immunotherapy efficacy and the clinical prognosis in advanced NSCLC patients.

## Materials and methods

### Study population

We included patients with stage IIIB or IV NSCLC who were treated with ICIs between September 2019 and July 2021 at the First Hospital of Shanxi Medical University. Pembrolizumab, nivolumab, camrelizumab and sintilimab were the different PD-1 inhibitors. The determination of the proportion of lymphocyte subsets is a routine test for patients with advanced lung cancer who require hospitalization for metronomic chemotherapy and immunotherapy, and is performed in almost all the patients on a voluntary basis as informed by the attending physician. By retrospectively reviewing the medical records of the patients, the data for peripheral blood lymphocyte subsets for different time-points, including, before any treatment, before immunotherapy or chemotherapy,and after 4 cycles of immunotherapy or chemotherapy were obtained.The inclusion criteria were as follows: (1) pathologically or cytologically diagnosed clinical stage IIIB or stage IV NSCLC; (2) Patients receiving first-line or non-first-line treatment with PD-1 inhibitors alone, or in combination with chemotherapy or anti-angiogenic drugs were included in the immunotherapy group, and patients receiving platinum-based chemotherapy with or without anti-angiogenic drugs were included in the chemotherapy group; (3) ECOG performance status between 0 and 2; and (4) availability of complete lymphocyte subsets and the follow-up data. Patients that met the following criteria were excluded: (1) suffered from other types of malignant tumors; (2) had acute infection, immunodeficiency and autoimmune disorders; and (3) received systemic corticosteroid treatment.

All the participants gave an informed consent. This study was approved by the Ethical Committee of The first Hospital of Shanxi Medical University (Ethics No. K125).

### Data collection and response assessment

The clinicopathological data collected for the analysis included gender, the age at diagnosis, smoking history, pathological type, stage, distant metastasis at first treatment, the treatment regimen, and the number of treatment lines.

The data collected from the laboratory included Epidermal growth factor receptor (EGFR) gene mutation status, PD-L1 expression level, proportion of peripheral blood lymphocytes (CD4^+^ T cells, CD8^+^ T cells, CD4^+^/CD8^+^ ratio, NK cells, Tregs, and B cells) before any treatment, before immunotherapy or chemotherapy, and after 4 cycles of immunotherapy or chemotherapy. In patients that received the first-line of treatment, the proportions of peripheral blood lymphocytes before any treatment and before immunotherapy or chemotherapy were the same.

The clinical efficacy of immunotherapy was evaluated based on the computed tomography (CT) or magnetic resonance imaging (MRI) of the patient tumors. The short-term clinical efficacy according to the solid tumor response evaluation criteria (RECIST) included complete response (CR), partial response (PR), stable disease (SD), and progressive disease (PD). Patients evaluated as CR, PR, and SD were classified as the effective group, and those evaluated as PD were classified as the ineffective group. The long-term efficacy was evaluated by the progression-free survival (PFS). PFS was the time from initiation of the treatment to the radiographic or clinical progression or death from any of the causes according to RECIST (RECIST-PFS).

### PD-L1 immunohistochemistry assessment

Appropriate amount of tumor tissue was obtained by percutaneous lung biopsy, thoracoscopic biopsy and bronchoscopy biopsy. Sections were stained with anti-PD-L1 antibodies (PD-L1 IHC 22C3 pharmDx, Dako) on the automatic immunohistochemical staining apparatus (DAKO Link 48).The PD- L1 tumor proportion score (TPS), which is the percentage of tumor cells showing partial or complete membrane staining, was divided into PD-L1 < 50% and PD-L1 ≥ 50%. The detailed operational procedures are provided in Additional file 2.

### Blood collection and flow cytometry

The proportion of the lymphocyte subsets in the peripheral blood were detected using BD FACSCanto II flow cytometry and flow antibodies. Three flow cytometry tubes were prepared for each peripheral blood specimen and labeled as Tube1, Tube2, and Tube3. CD45-PerCP-Cy5.5, CD3-FITC, CD4-APC, and CD8-PE were added to the tube1 to detect the proportion of CD4^+^ T cells (CD3+CD4+) and CD8^+^ T cells (CD3+CD8+) in the peripheral blood lymphocytes. Tube 2 was added to CD16+56-PE, CD45-PerCP-Cy5.5, CD3-FITC, and CD19-AP were added to the tube 2 to detect the proportion of NK cells (CD45+CD3-CD16+CD56+) and B cells (CD45+CD3-CD19+) in the peripheral blood lymphocytes. CD45-PerCP-Cy5.5, CD4-FITC, CD25-APC, and CD127-PE were added to tube 3 to detect the proportion of Tregs (CD45+CD4+CD25HICD127LOW) in the peripheral blood lymphocytes. Finally, CD4^+^/CD8^+^ ratio was calculated by dividing the proportion of CD4^+^ T cells by the proportion of CD8^+^ T cells. According to the flow cytometry detection platform in our hospital, the normal reference ranges for the above parameters were defined as follows: CD4^+^ T cells (30–50%), CD8^+^ T cells (20–35%), CD4^+^/CD8^+^ ratio (1–2), NK cells (20–35%), Tregs (3–7%), and B cells (5.6–16%). The detailed operational procedures and related materials are provided in Additional file 1.

### Statistical analysis

Quantitative data was expressed as mean ± standard deviation. T-test was adopted for comparison between groups, and paired t-tests were used to compare the before and after treatment groups. Qualitative data was expressed as the number of cases (%), and *X*^2^ test was also used for comparison between groups. In prognostic analysis, we used the median proportion of lymphocyte subsets as the threshold to define low and high levels. Prognostic multifactorial analysis was performed with logistic regression. The ROC curve was used to analyze the predictive value of T lymphocyte subsets as a biomarker for measuring the efficacy of immunotherapy NSCLC patients. Kaplan–Meier curve was drawn, and the Log Rank test was carried out to compare the differences in PFS between the different groups. Cox proportional hazard regression model was employed to analyze the risk of poor prognosis among different groups after other variables were calibrated. All statistical analyses were fulfilled with the support of SPSS software version 22.0. A *P* value < 0.05 was considered statistically significant.

## Results

### Patient characteristics

Records of a total of 276 patients were included in this study. Of these, 153 were in the immunotherapy group and 123 in the chemotherapy group.The clinical and pathological characteristics are reported in Table 1. EGFR gene mutations were detected by next-generation sequencing (NGS) in 161 participants, of which 65 participants were EGFR-sensitive mutation positive. EGFR gene mutation participants received ICIs after EGFR tyrosine kinase inhibitor (EGFR-TKI) treatment. Immunohistochemistry was used to test the protein expression levels of PD-L1 in 130 participants, of which 44 had TPS ≥ 50%.

**Table 1:** Clinical and pathological characteristics

Characteristics	N	%
Gender		
Male	191	69.2
Female	85	30.8
Age		
< 65	167	60.5
≥ 65	109	39.5
Smoker		
*No*	88	31.9
*Yes*	188	68.1
Histology		
Adenocarcinoma	140	50.7
Squamous cell carcinoma	136	49.3
Distant metastasis		
*No*	180	65.2
*Yes*	96	34.8
EGFR mutation		
*No*	96	59.6
*Yes*	65	40.4
PD-L1expression		
< 50%	86	66.2
≥ 50%	44	33.8
Treatment line		
Immunotherapy group		
First line	102	66.7
Second line and beyond	51	33.3
Chemotherapy group		
First line	96	78.0
Second line and beyond	27	22.0
Treatment model		
Immunotherapy group		
ICIs alone	22	14.4
Chemotherapy combined with ICIs	125	81.7
Anti-angiogenesis drugs combined with ICIs	6	3.9
Chemotherapy group		
Chemotherapy alone	115	93.5
Chemotherapy combined with Anti-angiogenesis drugs	8	6.5

### Factors influencing lymphocyte subsets

We collected lymphocyte subsets from patients with advanced NSCLC before any treatment and analyzed the impact of the clinicopathological features on the different lymphocyte subsets (Table 2). The proportion of CD4^+^ T cells (*P* = 0.027) was higher in females than in males. The proportion of NK cells was higher in males than in females (*P* = 0.007). Stage IV patients with distant metastasis had a higher proportion of Tregs (*P* = 0.015). Patients with EGFR mutations had lower CD8^+^ T cells proportion (*P* < 0.001) and higer CD4^+^/CD8^+^ ratio (*P* = 0.019). There was no effect of age, smoking, histology and PD-L1 expression on the lymphocyte subsets.

**Table 2:** The impact of clinical and pathological characteristics on lymphocyte subsets

	CD4^+^T	*P*	CD8^+^T	*P*	CD4^+^T/CD8^+^T	*P*	NK	*P*	Tregs	*P*	B	*P*
Gender		0.027		0.815		0.069		0.007		0.091		0.549
Male	34.35 ± 9.05		32.67 ± 9.44		1.15 ± 0.48		22.43 ± 11.85		6.77 ± 2.20		7.96 ± 4.06	
Female	36.96 ± 8.88		32.37 ± 10.4		1.27 ± 0.58		18.81 ± 9.45		6.24 ± 2.81		8.27 ± 3.82	
Age		0.168		0.902		0.852		0.163		0.199		0.580
< 65	35.76 ± 9.08		32.52 ± 9.12		1.19 ± 0.50		20.55 ± 10.82		6.76 ± 2.51		8.16 ± 3.70	
≥ 65	34.22 ± 8.99		32.67 ± 10.64		1.18 ± 0.55		22.49 ± 11.89		6.38 ± 2.23		7.88 ± 4.40	
Smoker		0.396		0.708		0.832		0.227		0.499		0.877
*No*	35.83 ± 8.58		32.90 ± 9.32		1.18 ± 0.49		20.21 ± 9.56		6.44 ± 2.97		8.11 ± 3.47	
*Yes*	34.84 ± 9.28		32.43 ± 9.94		1.19 ± 0.53		21.84 ± 11.98		6.68 ± 2.10		8.03 ± 4.21	
Histology		0.281		0.113		0.216		0.247		0.099		0.845
Adenocarcinoma	34.57 ± 8.42		33.49 ± 10.5		1.15 ± 0.52		20.54 ± 10.13		6.37 ± 2.23		8.01 ± 3.34	
Squamous cell carcinoma	35.75 ± 9.67		31.63 ± 8.80		1.23 ± 0.51		22.12 ± 12.34		6.85 ± 2.57		8.10 ± 4.56	
Distant metastasis		0.969		0.124		0.436		0.425		0.015		0.129
*No*	35.14 ± 9.10		31.92 ± 9.50		1.21 ± 0.51		21.71 ± 11.65		6.35 ± 2.40		8.29 ± 4.42	
*Yes*	35.18 ± 9.03		33.81 ± 10.09		1.15 ± 0.52		20.58 ± 10.56		7.09 ± 2.36		7.61 ± 2.95	
EGFR mutation		0.527		< 0.001		0.019		0.294		0.938		0.814
*No*	35.52 ± 8.30		34.75 ± 9.69		1.11 ± 0.40		20.23 ± 10.35		6.27 ± 1.97		8.13 ± 3.59	
*Yes*	34.61 ± 9.69		28.96 ± 10.17		1.32 ± 0.63		22.27 ± 13.04		6.24 ± 2.06		8.28 ± 4.25	
PD-L1 expression		0.873		0.986		0.743		0.476		0.404		0.454
< 50%	35.29 ± 9.71		33.05 ± 10.34		1.17 ± 0.57		20.46 ± 11.89		6.12 ± 1.81		7.69 ± 3.54	
≥ 50%	35.02 ± 7.72		33.02 ± 10.60		1.21 ± 0.51		18.98 ± 9.51		6.46 ± 2.37		8.21 ± 4.04	

### Changes in the proportion of lymphocyte subsets in different treatment groups

There were no statistical differences in the lymphocyte subsets between immunotherapy and chemotherapy groups before treatment. In the immunotherapy group, the proportions of CD4^+^ T cells (*P* < 0.001), CD4^+^/CD8^+^ ratio (*P* =0.012), NK cells (*P* = 0.002), and Tregs (*P* = 0.001) in the peripheral blood were significantly higher than baseline and the proportions of CD8^+^ T cells (*P* < 0.001) were significantly lower than the baseline. The proportions of CD4^+^ T cells (*P* < 0.001), CD8^+^ T cells (*P* < 0.001), Tregs (*P* < 0.001) and B cells (*P* = 0.002) in the peripheral blood were significantly lower in the chemotherapy group than in the baseline. CD4^+^ T cells, CD8^+^ T cells, CD4^+^/CD8^+^ ratio, NK cells and Tregs were higher in the immunotherapy group as compared to the chemotherapy group after treatment (Table 3).

**Table 3:** Changes in the proportion of lymphocyte subsets before and after treatment in different treatment groups

	Immunotherapy group			Chemotherapy group		
	Before	After	*P*	Before	After	*P*
CD4^+^	34.72 ± 9.50	39.34 ± 10.40	< 0.001	33.95 ± 8.40	23.95 ± 7.50^*^	< 0.001
CD8^+^	33.26 ± 10.9	26.64 ± 10.12	< 0.001	31.68 ± 9.26	23.16 ± 9.22^*^	< 0.001
CD4^+^/ CD8^+^	1.36 ± 2.19	1.83 ± 1.25	0.012	1.16 ± 0.47	1.23 ± 0.64^*^	0.158
NK	20.34 ± 10.45	22.60 ± 10.01	0.002	21.64 ± 10.71	20.06 ± 10.37^*^	0.052
Tregs	6.51 ± 1.87	7.21 ± 2.39	0.001	6.73 ± 2.54	5.55 ± 2.11^*^	< 0.001
B	8.27 ± 4.30	7.43 ± 4.52	0.117	8.00 ± 4.46	6.87 ± 3.02	0.002

Compared with the immunotherapy group, ^*^P < 0.05

### Comparison of the lymphocyte subsets in patients from different efficacy groups

**Fig. 1:**
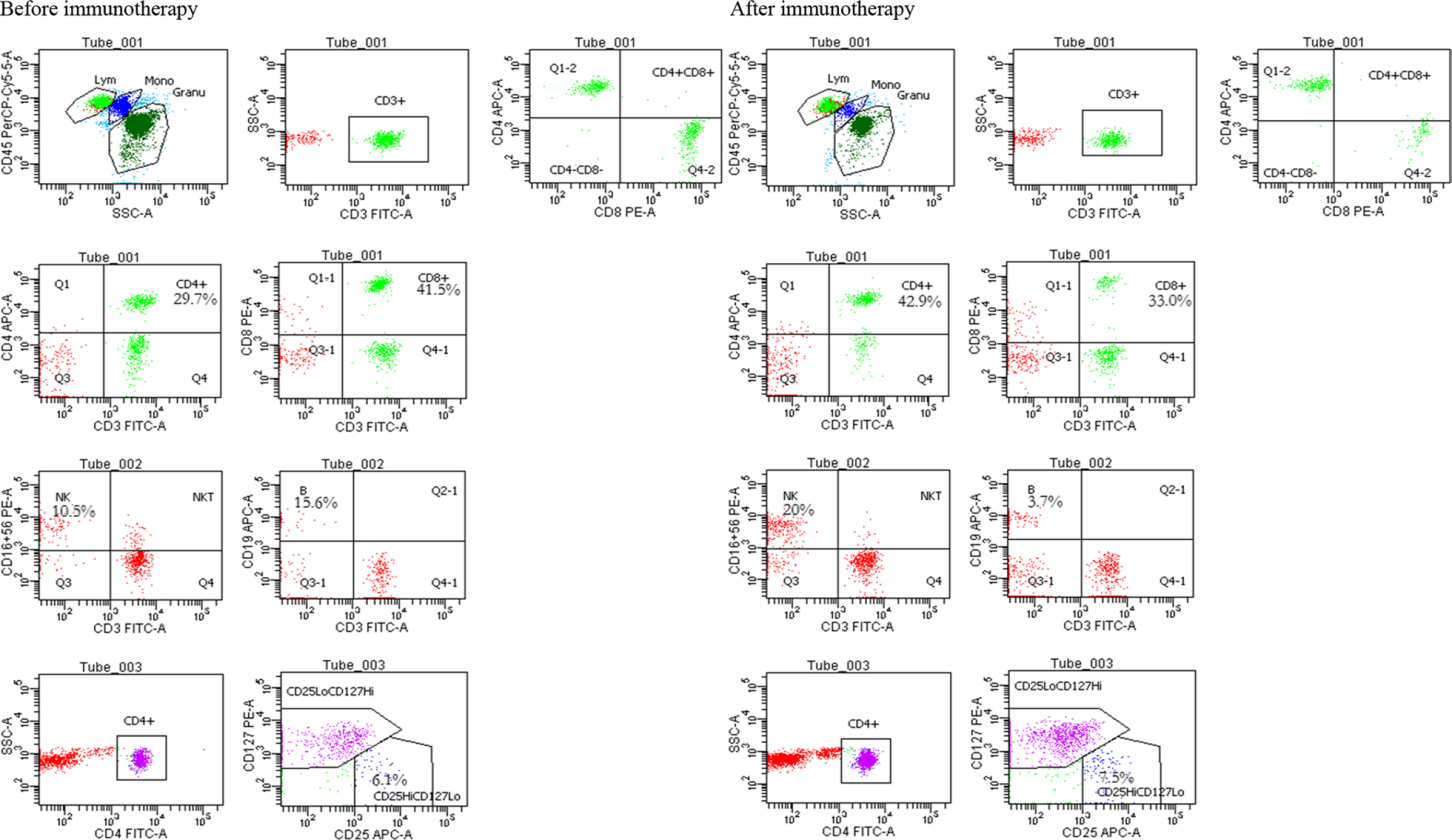
The flow image of a typical patient before and after immunotherapy

After 4 cycles of immunotherapy, out of 153 patients included in this study, 117 patients were classified as the effective group (CR 0, PR 48, SD 69) and 36 patients were classified as the ineffective group, with a treatment efficiency of 76.4%. The proportions of CD4^+^ T cells (*P* < 0.001), CD4^+^/CD8^+^ ratio (*P* = 0.010), NK cells (*P* = 0.003), and Tregs (*P* = 0.001) in the peripheral blood were significantly higher than the baseline, and the proportions of CD8^+^ T cells were significantly lower than the baseline for the effective group, as shown in Table 4. No significant differences were found regarding the proportion of CD4^+^ T cells (*P* = 0.486), CD8^+^ T cells (*P* = 0.425), CD4^+^/CD8^+^ ratio (*P* = 0.442), NK cells (*P* = 0.346), B cells (*P* = 0.630)  or Tregs (*P* = 0.394) in the peripheral blood of patients as compared to the baseline, for the ineffective group.The flow image of a typical patient before and after immunotherapy are shown in Fig. 1.

**Table 4:** Comparison of the different lymphocyte subsets before and after treatment in the immunotherapy effective and ineffective groups

	Effective group			Ineffective group		
	Before	After	*P*	Before	After	*P*
CD4^+^	35.78 ± 8.86	42.09 ± 9.46	< 0.001	31.27 ± 10.79	30.41 ± 8.12	0.486
CD8^+^	32.51 ± 10.51	23.63 ± 7.72	< 0.001	35.69 ± 11.93	36.44 ± 10.9	0.425
CD4^+^/CD8^+^	1.47 ± 2.48	2.10 ± 1.29	0.010	1.00 ± 0.48	0.95 ± 0.53	0.442
NK	21.28 ± 10.49	23.78 ± 9.05	0.003	17.30 ± 9.88	18.75 ± 12.01	0.346
Tregs	6.77 ± 1.81	7.61 ± 2.38	0.001	5.64 ± 1.82	5.93 ± 1.98	0.394
B	8.31 ± 4.19	7.36 ± 4.64	0.132	8.15 ± 4.68	7.65 ± 4.14	0.630

### Correlation between baseline lymphocyte subsets and immunotherapy efficacy

A total of 125 patients with complete data were evaluated after 4 cycles of immunotherapy, with 93 in the effective group (CR 0, PR 39, SD 54) and 32 in the ineffective group. Univariate analysis showed that the differences in PD-L1 expression, CD4^+^ T cells, NK cells, and Tregs were statistically significant between the two groups (*P* < 0.05). The proportion of PD-L1 expression (≥ 50%) was high in the effective group of immunotherapy, and the proportion of PD-L1 expression (< 50%) was high in the ineffective group. The proportion of baseline CD4^+^ T cells, NK cells and Tregs cells was higher in the effective group than in the ineffective group. We included these parameters into the logistic regression model and found that PD-L1 (OR: 0.315, 95% CI: 0.102–0.975, *P* = 0.045), CD4^+^ T cells (OR: 0.903, 95% CI: 0.853–0.957, *P* = 0.001), NK cells (OR: 0.913, 95% CI: 0.860–0.969, *P* = 0.003), and Tregs (OR: 0.645, 95% CI: 0.471–0.883, *P* = 0.006) were independent predictors of immunotherapy efficacy.

**Fig. 2:**
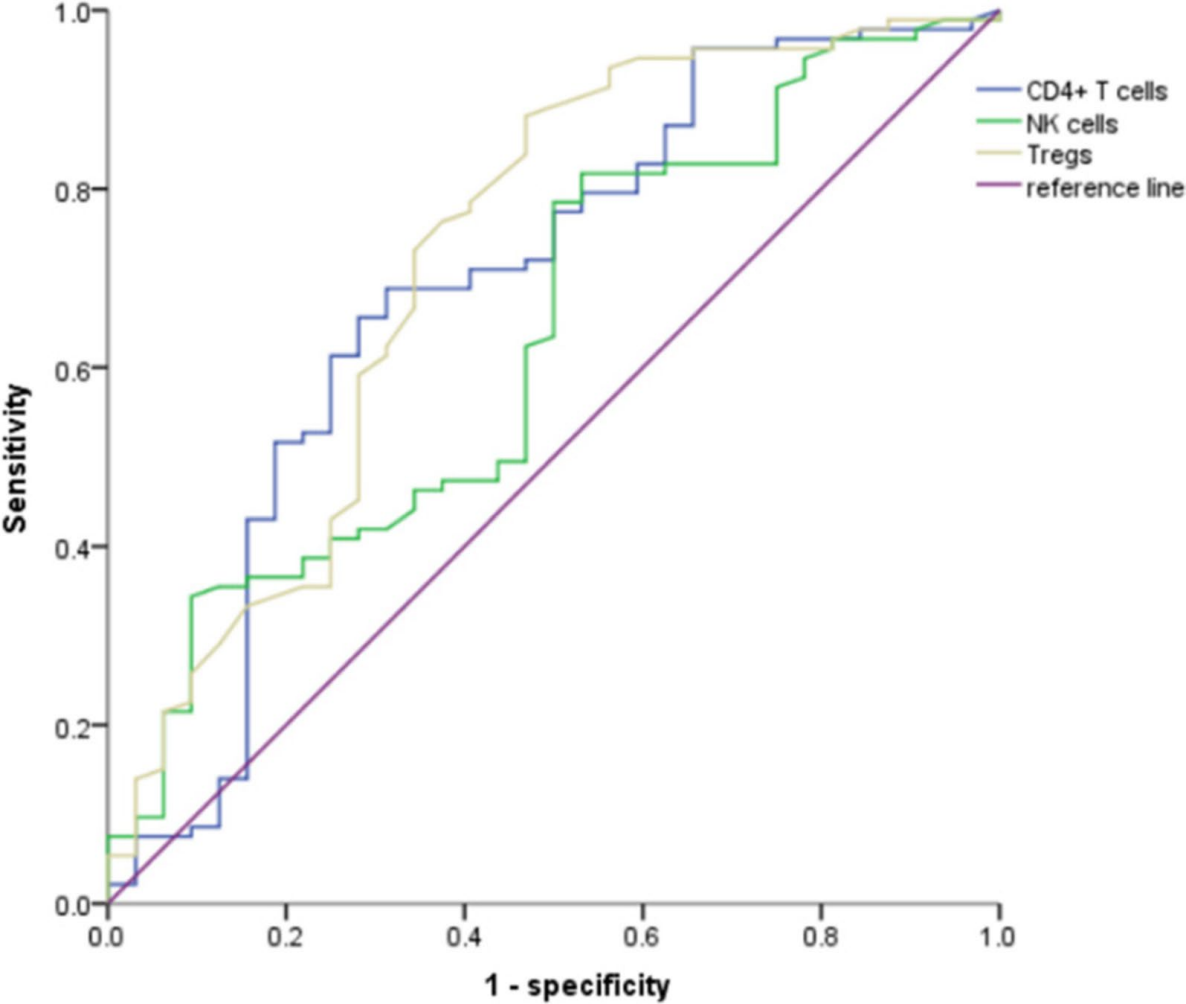
The ROC curve of CD4^+^ T cells, NK cells and Tregs before immunotherapy

In addition, our ROC working curves demonstrated that the AUC areas of baseline CD4^+^ T cells, NK cells, and Tregs predicted the efficacy of immunotherapy in NSCLC patients as 0.690, 0.634, and 0.722, respectively (Fig. 2). Corresponding parameter cut-off values for maximum AUC areas were calculated from the maximum Youden index, with a cut-off value of 33.450 for CD4^+^ T cells (sensitivity = 0.656, specificity = 0.719), 12.950 for NK cells (sensitivity = 0.817, specificity = 0.469), and 4.850 for Tregs (sensitivity = 0.882, specificity = 0.531).

### Correlation between the baseline lymphocyte subsets and PFS

**Fig. 3:**
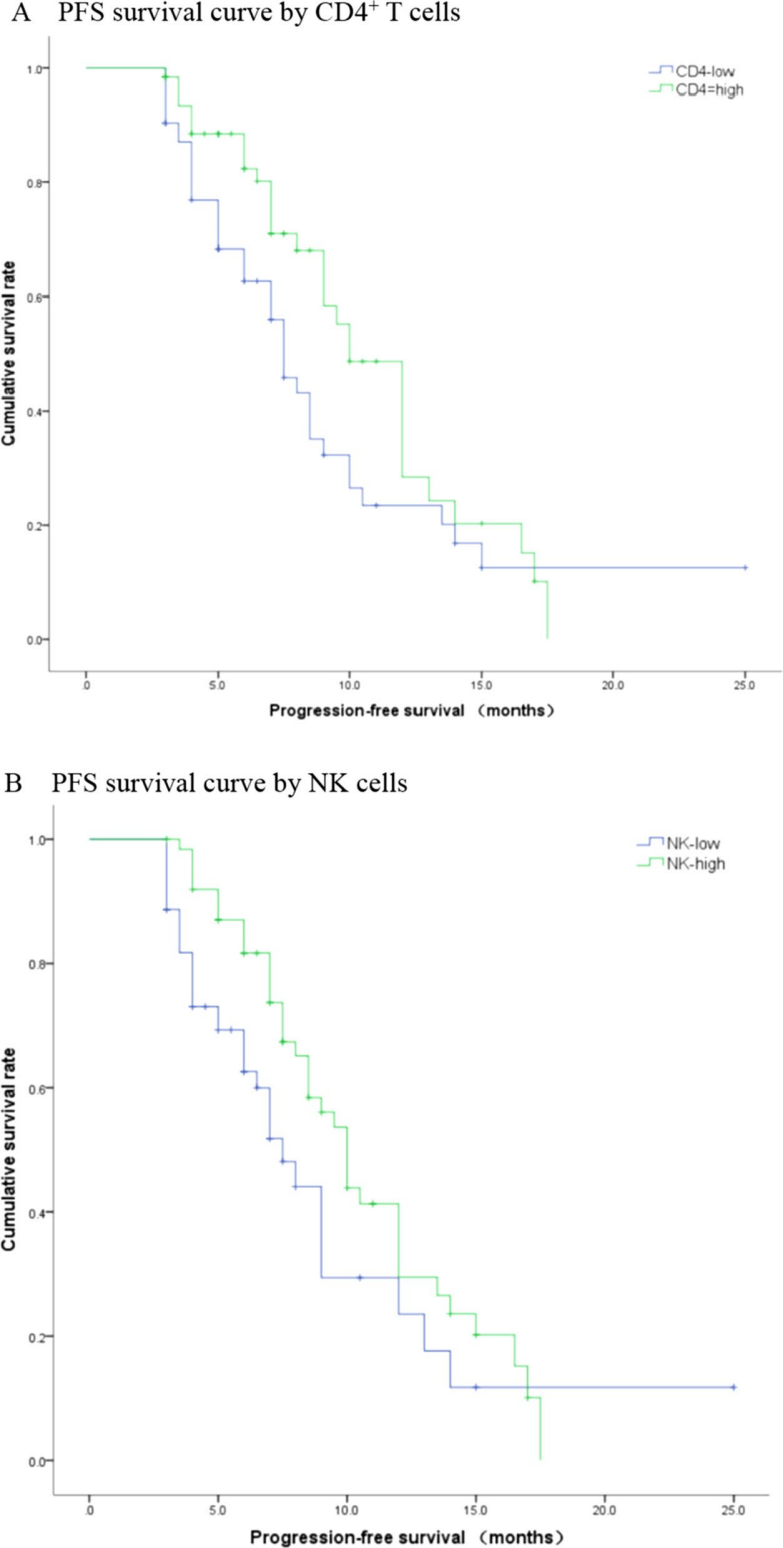
Kaplan–Meier survival curve of the correlation between the proportion of CD4^+^ T cell, NK cells and PFS

Among the 125 patients with NSCLC, the median PFS was 9 months (95% CI: 7.65–10.35). We used the median proportion of lymphocyte subsets as the threshold to define low and high levels. The proportion of CD4^+^ T cells was 34.30%, CD8^+^ T cells was 31.80%, CD4^+^/CD8^+^ ratio was 1.07, NK cells was 18.90%, Tregs was 6.10%, and B cells was 7.20%. Univariate analysis showed that gender, PD-L1 expression, CD4^+^ T cells, CD4^+^/CD8^+^ ratio and NK cells were associated with PFS. Age, smoking, pathology, distant metastasis, CD8^+^ T cells, Tregs, and B cells were not associated with PFS (Table 5). We performed multivariate Cox regression analysis factoring in the variables such as the gender, PD-L1 expression, CD4^+^ T cells, and NK cells. The results from these analyses revealed that PD-L1 expression (HR: 0.428, 95% CI: 0.245–0.748, *P* = 0.003), CD4^+^ T cells (HR: 0.454, 95% CI: 0.271–0.760, *P* = 0.003), and NK cells (HR: 0.491, 95% CI: 0.296–0.813, *P* = 0.006) were independent predictors of PFS. Kaplan–Meier survival curve analysis of the correlation between the proportion of CD4^+^ T cell, NK cells and PFS is shown in Fig. 3.

**Table 5:** Univariate analysis of the correlation between baseline lymphocyte subsets and PFS in 125 lung cancer participants

	PFS[Month, (95%CI)]	Log rank	*P*
Gender		3.862	0.049
Male	8.50 (7.02–9.98)		
Female	10.00 (6.71–13.29)		
Age		0.877	0.349
< 65	9.00 (7.44–10.56)		
≥ 65	8.50 (6.78–10.22)		
Smoker		1.481	0.244
*No*	9.00 (6.60–11.40)		
*Yes*	8.50 (7.53–9.47		
Histology		1.004	0.316
Adenocarcinoma	9.50 (8.21–10.80)		
Squamous cell carcinoma	7.50 (6.15–8.85)		
Distant metastasis		0.037	0.848
*No*	10.00 (7.43–12.58)		
*Yes*	8.50 (7.58–9.42)		
PD-L1 expression		5.034	0.025
< 50%	7.00 (6.68–10.32)		
≥ 50%	9.00 (7.00–13.01)		
CD4^+^ T		4.093	0.043
Low proportion group (< 34.30%)	7.50 (6.41–8.60)		
High proportion group (≥ 34.30%)	10.00 (8.48–11.52)		
CD8^+^ T		1.363	0.243
Low proportion group (< 31.80%)	10.00 (7.59–12.43)		
High proportion group (≥ 31.80%)	8.50 (7.08–9.92)		
CD4^+^/CD8^+^		4.126	0.042
Low ratio group (< 1.07)	8.00 (7.00–9.00)		
High ratio group (≥ 1.07)	12.00 (9.63–14.37)		
NK		4.212	0.040
Low proportion group (< 18.90%)	7.50 (6.08–8.92)		
High proportion group (≥ 18.90%)	10.00 (8.82–11.18)		
Tregs		2.783	0.095
Low proportion group (< 6.10%)	7.50 (6.63–8.37)		
High proportion group (≥ 6.10%)	10.00 (8.35–11.65)		
B		0.641	0.423
Low proportion group (< 7.20%)	10.00 (7.97–12.03)		
High proportion group (≥ 7.20%)	8.50 (7.33–9.67)		

Baseline represents the data obtained before immunotherapy

## Discussion

Although immunotherapy has achieved impressive results in the management of advanced NSCLC patients, reliable biomarkers for predicting therapeutic efficacy are still limited. Our current work focused on the changes in the composition of peripheral blood lymphocyte subsets before and after immunotherapy to assess their potential role as predictive biomarkers of immunotherapy efficacy and survival outcomes for patients with advanced NSCLC. The results showed that the proportions of CD4^+^ T cells, CD8^+^ T cells, Tregs and B cells decreased after chemotherapy as compared to the baseline. The proportions of CD4^+^ T cells, CD8^+^ T cells, CD4^+^/CD8^+^ ratio, NK cells and Tregs were higher after immunotherapy than after chemotherapy administration. Compared to the baseline, the proportions of CD4^+^ T cells, CD4^+^/CD8^+^ ratio, NK cells and Tregs in the peripheral blood of the immunotherapy effective group were significantly higher and the proportion of CD8^+^ T cells was significantly lower. In contrast, there were no statistically significant differences in these biomarkers in the ineffective group. Higher proportions of baseline CD4^+^ T cells and NK cells were associated with effective immunotherapy and a longer PFS, and higher proportions of baseline Tregs were associated with effective immunotherapy as well. The ROC curves further confirmed the predictive role of CD4^+^ T cells, NK cells, and Tregs on the efficacy of immunotherapy. Moreover, we analyzed the factors that could affect lymphocyte subsets, with the goal of determining the changes in the proportion of peripheral blood lymphocyte subsets with different clinicopathological characteristics. This would enable the identification of the population of patients that might benefit from immunotherapy. Results showed that NK cells were higher in males; CD4^+^ T cells were higher in females;the proportion of Tregs was higher in stage IV patients with distant metastasis; and the proportion of CD8^+^ T cells was lower and CD4^+^/CD8^+^ ratio was higher in patients with EGFR mutations. This is consistent with the findings of Mazzaschi et al. [12–14].

As an integral component of the anti-tumor immunity, peripheral blood CD4^+^ T cells help regulate and promote priming, migratory potential, and killing activity of cytotoxic T lymphocytes (CTLs) [15]. Our study showed that the proportion of CD4^+^ T cells increased in the patients within the effective immunotherapy group as compared to the baseline, while the change in CD4^+^ T cells in the ineffective group was not statistically significant. This suggests that patients in the effective group obtained a durable antitumor response, suggesting a continuous recruitment of antitumor T cells from the peripheral blood [16]. In addition, we found that higher levels of CD4^+^ T cells before immunotherapy were associated with better efficacy and a longer PFS. Similar conclusions were reached by previous studies. Ottonello et al. [17] found that high levels of CD4^+^ T cells were associated with a longer overall survival (OS) and PFS in advanced NSCLC patients treated with nivolumab. The value of certain CD4^+^ T cell subsets in predicting the efficacy of immunotherapy and patient survival has also been discovered now. A high proportion of memory CD4^+^T cells can be used to identify the clinical beneficiaries before the start of immunotherapy [18]; Hiroshi and colleagues identified a CD62Llow CD4^+^ T effector memory subset, and reported that the responders had significantly higher proportion of this effector subset prior to PD-1 blockade [15]. Another small-scale study in NSCLC patients treated with nivolumab showed that higher ratios of systemic central memory and effector subsets in the total populations of CD4^+^ T cells was associated with immunotherapy benefit [19]. A strong expansion of the highly differentiated CD28^−^ CD4^+^ T lymphocytes (CD4THD) was significantly associated with a hyper-progressive disease (HPD) [20]. In conclusion, CD4^+^ T cells play an important role in anti-tumor immunity, and dynamic monitoring of CD4^+^ T cells during immunotherapy could help to evaluate the prognosis of patients and screen for possible patient groups that might particularly benefit. In addition, Miren et al. found that PD-1/LAG-3 co-signaling induces dysfunctional proliferation of systemic CD4^+^ T cells, which in turn hampers the helper functions over peripheral CD8^+^ responses [18]. Combined blockade of PD1/LAG-3 may further improve the efficiency of immunotherapy in the future.

CD8^+^ T cells can expand and differentiate into cytotoxic T lymphocytes (CTL) that infiltrate tumors through peripheral blood migration and play an important role in antitumor immunity through the direct killing of tumor cells [21]. We found that the proportion of CD8^+^ T cells in the immunotherapy effective group was reduced as compared to the baseline, which is consistent with the findings of Minglei et al. [22]. This is in contradiction with the systemic expansion of CD8^+^ T cells after PD-1 blockade therapy in lung cancer patients [23]. We evaluated three explanations for this phenomenon. Firstly, patients with lung cancer treated with PD-1 inhibitors have a highly specific subset of proliferating CD8^+^ T cells in the peripheral blood, expressing effector-like phenotypes (HLA-DR+, CD38^+^, Bcl-2lo), costimulatory molecules (CD28, CD27, ICOS), and high levels of PD-1. Approximately 70% of the patients showed active proliferation of this CD8^+^T cells subset (PD-1+CD8^+^ T cells) after ICI treatment. This population was more likely to respond to PD-1 inhibitors and promote a longer PFS [24]. High baseline levels of PD-1+CD8^+^ T cells promotes a longer OS after nivolumab treatment in NSCLC patients [17, 25]. Secondly, the CD28/B7 pathway plays an important role in the proliferation of CD8^+^T cells after PD-1 blockade therapy in lung cancer patients. However, many human CD8^+^ TILs do not express CD28, which implies that they are less responsive to CD28 mediated proliferation of CD8^+^ T cells upon PD-1 blockade [26]. Thirdly, non-optimal timing of blood sampling might contribute as well. Induction of CD8^+^ T-cell response in the peripheral blood by blocking the PD-1 pathway is transient and detected only during the first 4 weeks after treatment initiation, however, these cells then migrate to the tumor sites [24]. Accordingly, the CD4^+^/CD8^+^ ratio was higher after immunotherapy than at the baseline. The CD4^+^/CD8^+^ ratio is a marker of cell-mediated immunity in cancer patients, and its reduction is related to a low immunological function [27]. This proves that the immunosuppression status of patients in the effective immunotherapy group was improved.

Toshiko's research in the previous years confirmed that the blockade of PD-L1 leads to the activation of NK cells and enhances the direct antitumor function of NK cells [28]. Myeong et al. also indicated that NK cell activity can be used as a biomarker to predict the response to immunotherapy in NSCLC patients [29]. Our research corroborated it in that the NK cells proportion increased in the patients in the effective immunotherapy group as compared to the baseline, and a higher baseline NK cells proportion is correlated with better efficacy and a longer PFS. This is consistent with the research done by Giulia and Peng et al. [12, 30]. In summary, the positive role of NK cells in tumor immune monitoring has been confirmed by more and more studies. Nowadays, medicines that modulate the proliferative activity and function of NK cells have been developed for the purpose of producing synergistic antitumor efficacy in combination with ICIs [31, 32]. Our results will further advance this field of research, as we show that higher baseline NK cell levels in patients with advanced NSCLC associates with improved immunotherapy efficacy.

Tregs play a crucial immunosuppressive role in patients with cancer. Our research showed that Tregs of patients in the clinical benefit group increased as compared to the baseline. This may be due to the Treg-NK interaction. Tregs selectively express membrane-bound transforming growth factor-β, down-regulate the expression of NKG2D on NK cells in vitro and in vivo and inhibit NK cells effector functions as a means of achieving immune homeostasis in organisms [33]. Giulia et al. also observed that following PD-1 blockade, the rise in NK cells in the clinical benefit group was accompanied by a significant increase in the number and proliferation of Tregs [12]. The current study also showed that a high Tregs proportion at baseline predicts a good immunotherapeutic response, in agreement with the findings of Jose et al. They observed an inverse correlation between the number of tumor Tregs and the number of peripheral Tregs in the mouse tumor model [34]. This means that ICIs treatment improves local immune microenvironment in the tumor. However, this study did not find a correlation between Tregs and long-term survival. Analyzing the role of circulating Treg subsets, Athanasios et al. reported for the first time that at the baseline, high levels of naive and effector Tregs were associated with a longer OS, while a high frequency of terminal effector Tregs was associated with a longer PFS and OS [35]. Jinyan et al. provided evidence that FOXA1+ Treg subsets promoted tumor growth and were associated with a poor response to lung cancer treatment [36]. Our next study will focus on the correlation between peripheral blood Treg subsets and long-term survival of patients treated with immunotherapy, in order to provide novel and reliable targets for inhibiting Tregs.

B cells activate T cells by antigen presentation [37] and the cytotoxic potential of activated B cells leads to the direct impairment of tumor development [38]. In this study, the proportion of peripheral blood B cells decreased after chemotherapy, suggesting humoral immunity was impaired. Johanna et al. reached similar conclusions to ours, and reported that 4–6 cycles of first-line chemotherapy reduced the absolute counts of B cells, independent of the subsets of B cells [16]. Ying et al. demonstrated that chemotherapy decreased the absolute counts of B cells, followed by a gradual increase, but ultimately did not reach the baseline level, suggesting that the effects of the chemotherapy on lymphocyte subsets is long-term [39]. There was no increase in the proportion of B cells after immune checkpoint inhibitor treatment. This is due to the fact that although B cells also express inhibitory surface markers such as PD-1, it is unclear whether PD-1 expression on B cells plays a role in the antitumor immune response or checkpoint inhibition therapy. In addition, several studies have revealed that B cells also play a pro-tumorigenic role, for example, the plasma-like B cells promote cancer cell growth in the advanced stage of NSCLC [40]. Also, B cells mediated suppression of activated CD8^+^ T cells that kill tumor cells in NSCLC patients upon ICI treatment, may decrease with age, implying that B cells may play a pro-tumorigenic role in younger NSCLC patients [18].

Previous studies have shown that chemotherapeutic agents not only kill tumor cells, but also damage the immune cells, and thus disturb the anti-tumor immune response [8, 10, 16]. Our study showed that CD4^+^ T cells, CD8^+^T cells, Tregs cells, and B cells were reduced after chemotherapy. A study by Wang et al. confirmed that proliferating T cells of all the subsets, were almost entirely depleted at day 8 following chemotherapy. Of these, Tregs were the most profoundly depleted cells at this time point [41]. This may be because paclitaxel significantly reduces all the subsets of Tregs [42]. There were no statistical differences in each of the parameters between the immunotherapy and chemotherapy groups before treatment. CD4^+^T cells, CD8^+^T cells, CD4^+^/CD8^+^, NK cells, and Tregs were all higher after immunotherapy than after chemotherapy, which implied that the overall immune status of patients was better after immunotherapy than after chemotherapy.

Although chemotherapy causes damage to immune cells, we need not worry that the anti-tumor effect of ICIs is diminished by the reduction of lymphocyte subsets in chemotherapy combination or sequential immunotherapy modalities. This is because, first of all, the reduction in lymphocyte subsets induced by chemotherapy is dynamic and transient [43]. Chemotherapy-induced increase in T cell proliferation, among the subsets of CD8^+^ T cells, and naive T cells, was the highest on day 6 after the treatment [44]. However, the increase in T-cell proliferation was not sufficient to restore T-cell numbers to pre-treatment levels [45]. Secondly, this proliferation of T cells is antigen-independent, termed as homeostatic repopulation. The homeostatic proliferation of T cells breaks tolerance, temporarily restoring the immune response to previously tolerated antigens [42]. Immune checkpoint inhibition can further augment anti-tumor immune responses by maintaining T cells in an activated state. Therefore, chemotherapy combined with immunotherapy or sequential immunotherapy may create a synergistic effect that augments anti-tumor immune responses [46].

We must acknowledge the limitations in our study. Firstly, our work did not obtain the absolute count of lymphocyte subsets. It would be more valuable to prospectively collect samples for analyzing the proportion and absolute count of lymphocyte subsets, in future studies. Furthermore, ICIs and treatment combinations obtained from the different pharmaceutical companies could have added further confounding factors into the analyses in our study. Finally, considering that the benefit to OS is the main goal of immunotherapy for lung cancer patients, future research must extend the observation time and treatment cycles, and monitor the changes in lymphocyte subsets longitudinally to explore their correlation with OS.

## Conclusions

In summary, our results indicate that immune checkpoint inhibitors induce changes in the proportion of peripheral blood lymphocyte subsets in patients with effective immunotherapy. High level of CD4^+^ T cells, NK cells and Tregs can predict a better immunotherapy efficacy in NSCLC patients, and a high level of CD4^+^ T cells and NK cells are associated with a longer PFS. Therefore, peripheral blood lymphocyte subsets may serve as valuable markers of therapeutic efficacy and prognosis for advanced NSCLC patients.

## Supplementary Information

**Additional file 1.** Flow cytometry staining and analysis.

**Additional file 2.** PD-L1 Immunohistochemistry.

## Data Availability

The datasets generated and /or analysed during the current study are not publicly available, but are available from the corresponding author on reasonable request.

## Abbreviations

NSCLC: Non-small cell lung cancer
ICIs: Immune checkpoint inhibitor
PFS: Progression-free survival
Tregs: Regulatory T cells
PD-L1: Programmed cell death ligand 1
TMB: Tumor mutation burden
LIPI: Lung immune prognostic index
TILs: Tumor-infiltrating lymphocytes
EGFR: Epidermal growth factor receptor
CT: Computed tomography
MRI: Magnetic resonance imaging
CR: Complete response
PR: Partial response
SD: Stable disease
PD: Disease progression
RECIST: The solid tumor response evaluation criteria
NGS: Next-generation sequencing
EGFR-TKI: EGFR tyrosine kinase inhibitor
CTLs: Cytotoxic T lymphocytes
