# Supplementary material for: Association of lymphocyte subsets with efficacy and prognosis of immune checkpoint inhibitor therapy in advanced non-small cell lung carcinoma: a retrospective study
Source: BMC Pulm Med. 2022 Apr 28;22:166. doi: 10.1186/s12890-022-01951-x (PMC9052648; doi:10.1186/s12890-022-01951-x)
Supplement: Supplementary file 1 — Additional file 1. Flow cytometry staining and analysis. [file 12890_2022_1951_MOESM1_ESM.docx]

**Supplement: Flow cytometry**

1. Label flow cytometry tubes 1, 2 and 3, add 100 μL of blood sample to each tube, then add 20ul of antibody (Tube 1 was added to CD45-PerCP-Cy5.5,CD3-FITC,CD4-APC,CD8-PE.Tube 2 was added to CD16+56-PE,CD45-PerCP-Cy5.5,CD3-FITC,CD19-AP.Tube 3 was added to CD45-PerCP-Cy5.5,CD4-FITC,CD25-APC,CD127-PE) according to the antibody instructions(Table S1), add 100ul of anticoagulated peripheral blood to each tube and incubate for 20-30 minutes at room temperature;
2. After staining, add 1 mL of erythrocyte lysate to each tube. Vibrate and leave at room temperature for 10 minutes in the dark, and centrifuge at 300g for 5 minutes;
3. The supernatant was discarded, 1 ml of PBS was added to each tube, washed once, and 50 ul of PBS was added to each tube, and the cell subsets proportion was detected by flow cytometry;
4. Data analysis with MULTISET software.

Table S1

| Anti- bodies Companies Product No. |
| --- |
| CD4^+^ T cells,CD8^+^ T cells  CD45-PerCP-Cy5-5 Beijing Tongsheng Shidai Biotechnology Co. Z6410002  CD3-FITC Beijing Tongsheng Shidai Biotechnology Co. Z6410002  CD4-APC Beijing Tongsheng Shidai Biotechnology Co. Z6410002  CD8-PE Beijing Tongsheng Shidai Biotechnology Co. Z6410002  NK cells,B cells  CD16+56-PE Beijing Tongsheng Shidai Biotechnology Co. Z6410010  CD45-PerCP-Cy5.5 Beijing Tongsheng Shidai Biotechnology Co. Z6410010  CD3-FITC Beijing Tongsheng Shidai Biotechnology Co. Z6410010  CD19-APC Beijing Tongsheng Shidai Biotechnology Co. Z6410010  Tregs  CD45-PerCP-Cy5.5 Beijing Tongsheng Shidai Biotechnology Co. Z6410010  CD4-FITC Beijing Tongsheng Shidai Biotechnology Co. Z6410005  CD25-APC Beijing Tongsheng Shidai Biotechnology Co. Z6410045-100T  CD127-PE Beijing Tongsheng Shidai Biotechnology Co. Z6410046-100T |
