# Supplementary material for: Association of lymphocyte subsets with efficacy and prognosis of immune checkpoint inhibitor therapy in advanced non-small cell lung carcinoma: a retrospective study
Source: BMC Pulm Med. 2022 Apr 28;22:166. doi: 10.1186/s12890-022-01951-x (PMC9052648; doi:10.1186/s12890-022-01951-x)
Supplement: Supplementary file 2 — Additional file 2. PD-L1 Immunohistochemistry. [file 12890_2022_1951_MOESM2_ESM.docx]

**Supplement: PD-L1 Immunohistochemistry**

1. The tissue specimens were fixed in 10% neutral formalin for 6h-24h, and the specimens were routinely taken and processed to make paraffin tissue;
2. Regular sections were dewaxed and dehydrated, repaired by the restorer for 40 min, washed in buffer, and the slides were placed in an automatic immunohistochemical staining machine and stained with anti-PD-L1 antibody;
3. scanning all immunohistochemical sections with a digital pathology section scanner and having two experienced pathologists interpret the scanned sections;
4. TPS was used as the expression result according to DAKO PD-L1 IHC 22C3 interpretation criteria.
